# A Case of Self-treatment Induced Recurrent Fixed Drug Eruptions Associated with the Use of Different Fixed Dose Combinations of Fluoroquinolone-Nitroimidazole

**Published:** 2014-11

**Authors:** Agnik Pal, Sukanta Sen, Suvajit Das, Arunava Biswas, Santanu K Tripathi

**Affiliations:** Department of Clinical and Experimental Pharmacology, Calcutta School of Tropical Medicine, Kolkata, West Bengal, India

**Keywords:** Self-medication, Drug combinations, Drug eruptions, Fluoroquinolones, Nitroimidazoles

## Abstract

A young male patient used fixed dose combinations of different fluoroquinolones and nitroimidazoles several times in the last few years for self-treating repeated episodes of diarrhea and loose motion. Each time, he experienced fixed drug eruptions that increased in number and severity on subsequent occasions. Suspecting association between the drug and the rashes, the patient each time discontinued the treatment prematurely, and preferred to switch to a similar formulation next time, but with different molecules of fluoroquinolone (ciprofloxacin or ofloxacin) and nitroimidazole (tinidazole or ornidazole). He could not however avoid the rash. This time the patient presented with multiple, round-to-oval, well-defined, hyperpigmented cutaneous patches of different dimensions, all over the body. He appeared to have run out of options and therefore consulted us seeking advice on how he should treat himself next time he suffered from diarrhea. Causality assessment by Naranjo’s algorithm revealed a definite relationship between the cutaneous adverse reaction and the offending drug. He was counselled regarding medication in general and advised, in particular, to avoid the tendency to self-treat any future episode of diarrhea.

## Introduction

Fixed drug eruptions (FDE) are a distinct type of drug eruptions that appear as pruritic, well circumscribed, round or oval-shaped, erythematous macules or edematous plaques, and characteristically recur at the same sites upon re-exposure to the offending drug. They usually resolve spontaneously with hyperpigmentation.^1^ The lesions after healing remain quiescent and present on the skin, mucous membrane, or on both for prolonged periods as gray-brown macules or plaques. Their number and severity may increase with repeated exposure. Swelling and redness of the skin is typically seen within 30 minutes to 8 hours after suspected drug exposure. Lesions are more commonly seen on extremities, genitals and perianal areas, but they may appear on any location.^1^^,^^2^

FDEs are not uncommon and are seen with a host of drugs, including nitroimidazoles^2^^,^^3^ fluoroquinolones^4^^,^^5^ and cross sensitivity and poly-sensitivity among different members of the same pharmacological class do exist.^2^^,^^6^ It is generally believed that if an individual develops FDEs to a particular drug, exposure to structurally similar drugs from the same pharmacological group should preferably be avoided.^2^

Diarrheal disorders are quite common in all age groups^7^ and are mostly of infective origin.^8^ The tendency to self-treat episodes of diarrhea among adults seems to be widespread,^9^ and people often indulge in self medication. This is not surprising, particularly in the backdrop of the irrational dispensing practices that prevails in India allowing easy availability of prescription medicines without a prescription. Different fixed dose combinations (FDC) consisting of an antiprotozoal and an antibacterial, are marketed in India for the treatment of diarrhea. While there is little evidence to justify the rationale for their use in diarrhea, they certainly expose the patients to higher risks of adverse reactions and increase emergence of drug resistance.^8^

Here we present a case of self-treatment induced repeated episodes of recurrent fixed drug eruptions secondary to the use of different fixed dose combinations of fluoroquinolone-nitroimidazole. 

## Case Presentation

**Figure 1:**
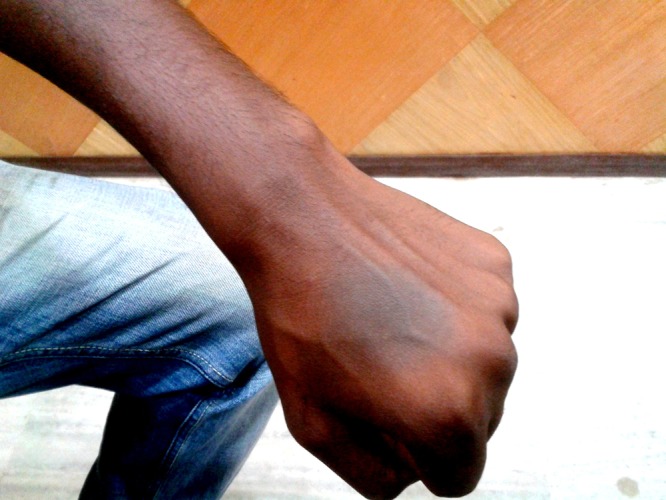
This figure shows the presence of fixed drug eruption in the dorsum of left hand.

**Figure 2:**
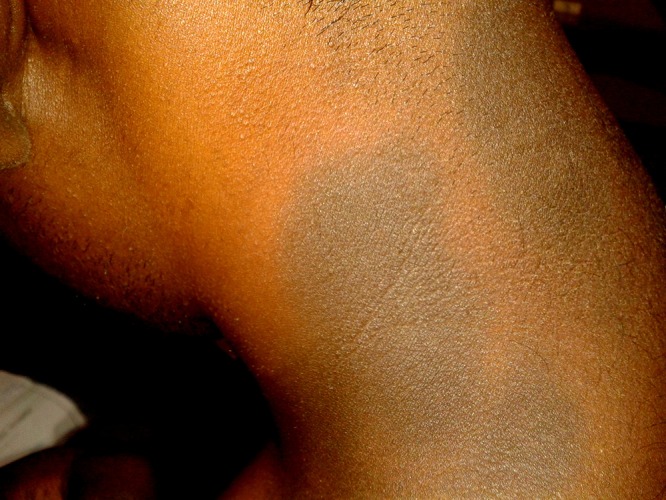
This figure shows the presence of fixed drug eruption in the back side of neck region.

**Figure 3:**
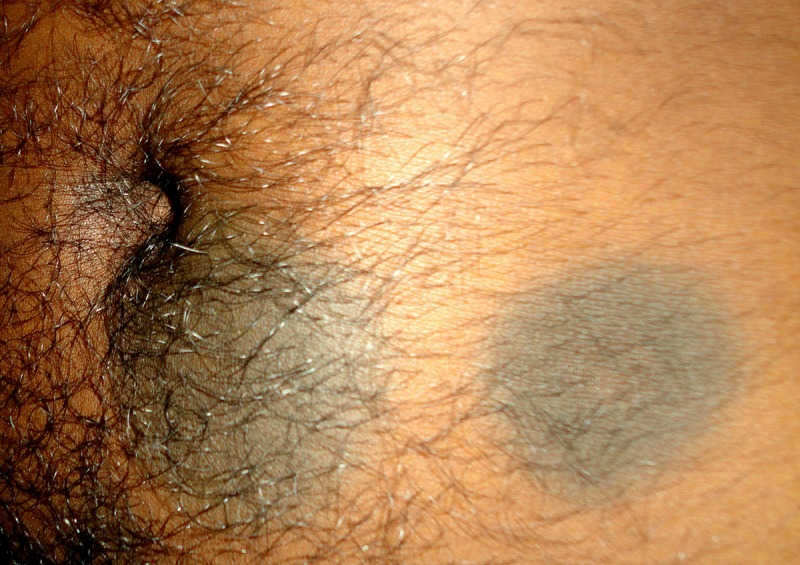
This figure shows the presence of fixed drug eruption in the abdomen.

**Figure 4:**
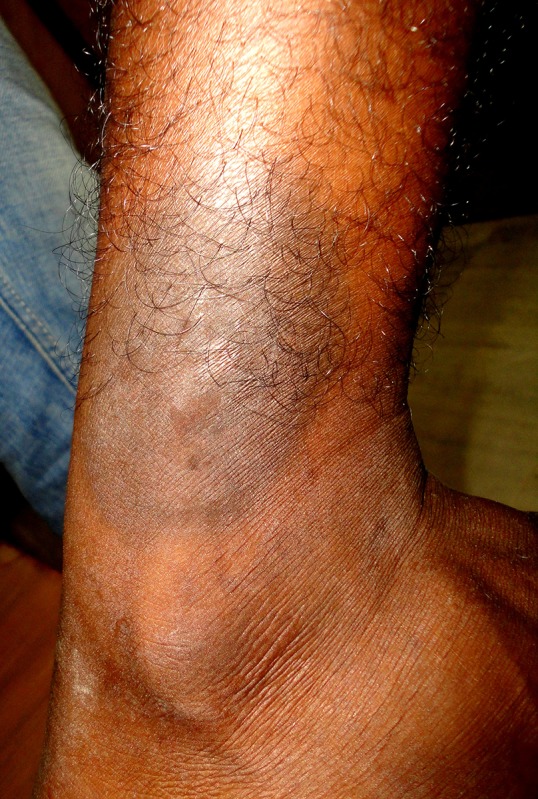
This figure shows the presence of fixed drug eruption in the lateral aspect of right leg just above lateral malleolus.

A 23-year-old male, college student, presented with multiple, round-to-oval, well-defined, hyperpigmented cutaneous patches of different dimensions all over the body, particularly more on the neck, trunk, forearms, and dorsum of the hands and legs (figures 1-4). Some of these lesions developed about one month back when he had taken an FDC of ciprofloxacin (500 mg) and tinidazole (600 mg) for acute gastroenteritis. Within 30 minutes of intake of the first dose, multiple vesicular lesions started to appear all over the body that were intensely itchy, and that on scratching turned within a few hours into fluid-filled purplish vesicles with burning sensation. He remained afebrile with no other major complaint. He took cetirizine (10 mg) for one week. The lesions gradually healed up in the next 10 days, leaving behind dark grey hyperpigmented lesions, which persisted at the time of his visit to us. The rest of the similar dark patches with which the patient presented were, as the history revealed, sequelae of exposure to FDCs of different fluoroquinolones and nitroimidazoles several times in the past few years. The medically lay patient indulged in self-medication whenever he suffered loose motion or diarrhea and he preferred taking similar oral FDCs combining a fluoroquinolone and a nitroimidazole. He experienced recurring episodes of similar cutaneous reactions each time he consumed such FDCs, on five such occasions (including the current one) in the last 2-3 years. Interestingly, each time he changed the molecules of the FDC (with ciprofloxacin or ofloxacin as fluoroquinolone, and tinidazole or ornidazole as nitroimidazole), expecting to avoid the cutaneous reaction. But, rather he experienced an increase in the number of sites and in size of the cutaneous lesions with repeated exposure to the qualitatively similar FDCs. As soon as the rashes appeared, he discontinued the treatment. Further probing revealed that sometimes he used only metronidazole instead of a FDC and there was no cutaneous reaction.

In this patient, there was a clear temporal association between the drug exposure and the episodes of FDEs and no other co-morbidity could have been implicated for the cutaneous lesions. He had no history of asthma or other allergic diseases. Neither relevant family history was present. Regarding the medical history and use of other drugs, nothing relevant could be elicited. Causality assessment by Naranjo’s algorithm^10^ revealed a definite relationship, with a score of nine, between the cutaneous adverse reaction and the offending drug. We intended to undertake a lymphocyte stimulation test for definitive diagnosis of drug allergy, but the patient did not agree to comply.

We counselled the patient regarding responsible use of medications in general and advised him not to indulge in self-treatment of any future episodes of diarrhea. 

## Discussion

Self medication has been defined as the use of drugs to treat self diagnosed disorders or symptoms or the intermittent, or continued use of prescribed drugs for chronic or recurrent disease or symptoms.^11^ This is a unique case of repeated self-treatment by a medically lay patient, but otherwise well-educated adult, despite recurrent episodes of FDEs.

The first reported case of fixed drug eruption by Bourns in 1889, described a series of sharply circumscribed, hyperpigmented lesions on the lips and tongue in a patient who had ingested antipyrine. Later, the French word “eruption erythemato-pigmentee fixe”, was coined by Brocq to denote this type of lesion, which meant “fixed drug eruption”.^4^

FDEs occurs more rapidly in patients who are intermittently receiving the causative drugs rather than those continuously receiving them; allowing the period required for sensitization. The sites involved earlier do not necessarily flare with each exposure.^12^ The drugs, mostly reported to cause FDE include ACE inhibitors, allopurinol, co-trimoxazole, sulfonamides, tetracyclines, cephalosporins, penicillin, clindamycin, trimethoprim, metronidazole, barbiturates, benzodiazepines, calcium channel blockers, carbamazepine, dextromethorphan, diltiazem, fuconazole, lamotrigine, NSAIDs, including aspirin, paclitaxel, paracetamol, phenolphthalein, proton pump inhibitors: omeprazole, lansoprazole, quinine salicylates and terbinafine.^13^

FDE is a form of classic delayed-type hypersensitivity mediated by CD8+T cells. Localized tissue damage is contributed mostly by intraepidermal CD8+T cells present in the lesions.^12^ However, FDE lesions usually appear within 2 hours of clinical challenge with the causative drug due to activation of mast cells localized in the vicinity of the epidermis in FDE lesions upon skin exposure to the causative drug.^1^

In this case, the patient on several occasions took different FDCs of fluoroquinolone and nitroimidazole without consulting a doctor, for treating diarrhea. It seems quite common in India that for trivial and for not-so-serious ailments, patients indulge in self-treating with polypharmacy or FDCs. Procuring these prescription-only medicines from retail pharmacies, without a valid prescription, is also not uncommon. However, when such patients repeat similar treatments and subsequently encounter similar adverse events, it becomes difficult, without perhaps employing specific objective tests, to identify the offender(s) as well as to rule out the possibility of cross sensitivity and polysensitivity.^1^^,^^14^ In the present case, the patient declined to subject himself to further challenge or any objective tests. Thus we can only presume that he might have sensitivity to any of the components of the different FDCs he consumed, namely tinidazole or ornidazole (but not to metronidazole), and ciprofloxacin or ofloxacin. Cross-sensitivity for FDEs among different nitroimidazoles and fluoroquinolones are well documented. There exists some selectivity as well; though belonging to the same pharmacological class and having a similar structure, some members have caused FDEs while others have been well tolerated. The difference in sensitivity can be attributed to the different side chains in their structure, for example, metronidazole has ethanol, tinidazole has ethylsulphonyl ethyl, and ornidazole has chloropropanol.^2^ FDEs seen with a drug is often found to be attributable to the chemical class it belongs. Therefore, it is advisable to avoid other drugs of the same class.^2^

Recurrence of FDEs upon self-treatment with FDCs of a fluoroquinolone and a nitroimidazole, to the best of our knowledge, has been documented in only one published case report,^15^ showing FDEs due to an FDC with ofloxacin and ornidazole. However, in our case, the patient, in order to self-treat repeated episodes of diarrhea and loose motion, used fluoroquinolone-nitroimidazole FDC products on several occasions in the last few years, each time with changing compositions (i.e. with a different fluoroquinolone and a different nitroimidazole) and each time he experienced FDEs in increasing number and severity. In fact, the patient himself suspected the association between the FDE and the FDC that not only prompted him to discontinue the treatment prematurely but also made him use a different FDC (i.e. with different molecules of fluoroquinolone and nitroimidazole) next time. Finally, he appeared to have run out of options and therefore consulted us seeking advice on how he should treat himself next time he suffers from diarrhea. The patient was counselled and appropriately advised on how he should conduct during any future diarrheal episodes he suffers.

Rampant irrational use of antimicrobial fixed dose combinations for common ailments like diarrhea and loose stool without medical guidance may result in a greater probability of inappropriate, incorrect or undue therapy. This may also result in increasing microbial resistance, more incidences of adverse drug reactions, increased morbidity and mortality. Patients gather information regarding medication from family members, peers as well as from advertisements, and indulge in self-treatment. In India, it is very common to notice the ill-informed self-medication practice, which poses an emerging challenge to health care providers. At the community level, such under-informed self-medication practices can increase the the burden of drug-induced diseases and thus encourage wasteful public expenditure.^16^

## Conclusion

FDEs are a group of characteristic adverse cutaneous drug reaction that appears to be largely unreported due to the indifference of patients as well as health care providers. FDEs due to FDCs of different fluoroquinolones and nitroimidazoles are rare and may seem interesting to medical fraternity. Besides, this report highlights peculiar self-medication behaviour of the patient that is worth sharing. This reiterates the importance of physicians’ role in patient counselling and patient education. This also underscores the importance of eliciting medication allergy history before prescribing. Besides, improved knowledge and understanding about self-medication may result in rational use and thereby limit adverse drug reactions of drugs. 

## Consent

Written informed consent was obtained from the patient for the publication of this case report and the accompanying images. A copy of the written consent is available for review by the Editor-in-Chief of this journal. 
